# MRI-Tracking of Dental Pulp Stem Cells In Vitro and In Vivo Using Dextran-Coated Superparamagnetic Iron Oxide Nanoparticles

**DOI:** 10.3390/jcm8091418

**Published:** 2019-09-09

**Authors:** Shahrokh Zare, Davood Mehrabani, Reza Jalli, Mahdi Saeedi Moghadam, Navid Manafi, Golshid Mehrabani, Iman Jamhiri, Samad Ahadian

**Affiliations:** 1 Stem Cell Technology Research Center, Shiraz University of Medical Sciences, Shiraz, Fars 71348-14336, Iran (S.Z.) (I.J.); 2 Department of Biochemistry, School of Biotechnology and Agriculture, Shiraz Branch, Islamic Azad University, Shiraz, Fars 71987-74731, Iran; 3 Burn and Wound Healing Research Center, Shiraz University of Medical Sciences, Shiraz, Fars 71987-74731, Iran; 4 Comparative and Experimental Medicine Center, Shiraz University of Medical Sciences, Shiraz, Fars 71348-14336, Iran; 5 Department of Laboratory Medicine and Pathology, University of Alberta, Edmonton, AB T6G 2R3, Canada; 6 Medical Imaging Research Center, Department of Radiology, Shiraz University of Medical Sciences, Shiraz, Fars 71348-14336, Iran (R.J.) (M.S.M.); 7 School of Medicine, Shahid Beheshti University of Medical Sciences, Tehran, Tehran 14348-75451, Iran; 8 Henry M. Goldman School of Dental Medicine, Boston University, Boston, MA 02118, USA; 9 Department of Bioengineering, University of California-Los Angeles, Los Angeles, CA 90049, USA

**Keywords:** MRI, tracking, labeling, dental pulp stem cells, iron oxide, nanoparticle

## Abstract

The aim of this study was to track dental pulp stem cells (DPSCs) labeled with dextran-coated superparamagnetic iron oxide nanoparticles (SPIONs) using magnetic resonance imaging (MRI). Dental pulp was isolated from male Sprague Dawley rats and cultured in Dulbecco’s modified Eagle’s medium F12 (DMEM-F12) and 10% fetal bovine serum. Effects of SPIONs on morphology, viability, apoptosis, stemness, and osteogenic and adipogenic differentiation of DPSCs were assessed. Prussian blue staining and MRI were conducted to determine in vitro efficiency of SPIONs uptake by the cells. Both non-labeled and labeled DPSCs were adherent to culture plates and showed spindle-shape morphologies, respectively. They were positive for osteogenic and adipogenic induction and expression of cluster of differentiation (CD) 73 and CD90 biomarkers, but negative for expression of CD34 and CD45 biomarkers. The SPIONs were non-toxic and did not induce apoptosis in doses less than 25 mg/mL. Internalization of the SPIONs within the DPSCs was confirmed by Prussian blue staining and MRI. Our findings revealed that the MRI-based method could successfully monitor DPSCs labeled with dextran-coated SPIONs without any significant effect on osteogenic and adipogenic differentiation, viability, and stemness of DPSCs. We provided the in vitro evidence supporting the feasibility of an MRI-based method to monitor DPSCs labeled with SPIONs without any significant reduction in viability, proliferation, and differentiation properties of labeled cells, showing that internalization of SPIONs within DPSCs were not toxic at doses less than 25 mg/mL. In general, the SPION labeling does not seem to impair cell survival or differentiation. SPIONs are biocompatible, easily available, and cost effective, opening a new avenue in stem cell labeling in regenerative medicine.

## 1. Introduction

Organ shortage has resulted in emergence of regenerative and personalized medicine with the aim of tissue replacement or enhancement in tissue function [1,2]. Among stem cells used for cell transplantation, adult mesenchymal stromal cells (MSCs) have emerged as a major source with several pre-clinical and clinical applications. MSCs have self-renewal, differentiation, migratory, anti-inflammatory, and immunosuppressive characteristics, which are required for cell-based therapies [3]. They can be isolated from various tissues, such as bone marrow [4], adipose [5], endometrium [6], umbilical cord blood [7], and dental pulp [8].

Among MSCs, dental pulp stem cells (DPSCs) resident within the perivascular niche of the dental pulp have high plasticity and multipotential characteristics. They can be isolated from tooth extractions in dental clinics non-invasively and have a heterogeneous cell population making them a suitable candidate for cell-based therapies and regenerative medicine [8]. The safety and feasibility of DPSCs were previously clarified by several authors [9], and they were shown to have a great potential for anti-inflammatory and regenerative applications [10].

Little information is available on events occurring after cell transplantation into the body. Therefore, there is a specific need to monitor transplanted cells within the body and quantify cell accumulation at the target site [11] to reveal mechanisms of cell behavior and function in tissue repair [12].

Magnetic resonance imaging (MRI) has been introduced as an excellent non-invasive method of cell tracking [12], which produces images with high spatial resolution in a non-ionizing and non-invasive manner [13]. MRI contrast agents have an important role in successful use of MRI in clinics. There has been a significant rise in the number of contrast-enhanced agents since introduction of the first MRI contrast agent gadolinium-diethylenetriamine pentaacetic (Magnevist, Schering AG) [14]. MRI contrast agents contain paramagnetic or superparamagnetic metal ions, influencing MRI signal properties of the surrounding tissue and increasing the sensitivity of MRI to detect several pathological processes [14].

One of the most popular contrast agents is superparamagnetic iron oxide nanoparticles (SPIONs) that are small crystalline magnetite structures (5–150 nm), which are easily available and biocompatible and can easily be endocytosed into the cell. These particles can also be imaged using transmission electron microscopy [15]. SPIONs consist of iron oxide nanocrystals coated with dextran or carboxy dextran, which have been clinically approved [14]. They are used in cell labeling in conjunction with MRI for tracking transplanted cells both in vivo and in vitro [11]. The dextran-coated SPIONs can affect the local magnetic field and can be visualized as a lack of signal with MRI [16].

Major effect of SPIONs is on T2 relaxation. Therefore, MR imaging is usually conducted using T2/T2-weighted sequences in which the tissue signal loss is because of the susceptibility effects of the iron oxide core. Enhancement on T1-weighted images can also be observed with smaller sizes of SPIONs. In 2018, superparamagnetic particles of iron oxide were used to detect lymph node metastases. The signal loss could be observed in normal lymph nodes; however, in the areas that the lymph nodes were replaced by tumoral cells, less signal loss was visible due to less uptake of particles of iron oxide. The signal loss could be noted in the areas where iron oxide was presented [17]. SPIONs also enable various approaches, such as stem cell isolation, cell differentiation, cell patterning, and gene transfer. They are also used in vitro for scaffold-free association of 3D cell-laden constructs [18]. We hypothesized that exogenous DPSCs labeled with SPIONs may allow for monitoring of the localization and retention of cells via MRI without negatively affecting cell survival or differentiation. Therefore, this study aims to use DPSCs labeled with SPIONs and coated with dextran to be traced by MRI, while the effect of SPIONs on viability, stemness, and differentiation of DPSCs was also detailed.

## 2. Subjects and Methods

### 2.1. Cell Isolation

For isolation of DPSCs, five male Sprague Dawley rats (4–6 weeks old, 180–220 g) were purchased from the Animal House of Shiraz University of Medical Sciences. To isolate the cells, the animals were euthanized and sacrificed under anesthesia using 50 mg/kg of 10% ketamine (Alfasan, Woerden, The Netherlands) and 8 mg/kg of 2% xylazine (Alfasan, Woerden, The Netherlands). The maxilla and mandible were separated and transferred into a 50 mL tube containing phosphate-buffered saline (PBS, Sigma-Aldrich, St. Louis, USA) containing 3% penicillin-streptomycin (Sigma-Aldrich, St. Louis, USA). All experimental protocols were approved by the Ethics Committee of Shiraz University of Medical Sciences and all experiments were conducted in accordance with the approved guidelines of Iran Veterinary Organization.

### 2.2. Cell Culture

Under a laminar flow hood in an aseptic condition, the connection between bone and teeth were cut and, by using a 21 G needle attached to a 1 mL syringe filled with Dulbecco’s modified Eagle’s medium F12 (DMEM-F12, Gibco, Waltham, USA), the dental pulp tissue was taken out and transferred into a 15 mL tube containing DMEM-F12. The dental pulp tissue was washed in sterile PBS three times and the intact pulp tissue was mechanically cut into small pieces by a sterile blade. The chopped tissue was then transferred into a 15 mL tube containing 5 mL of DMEM-F12.

The tube content was then centrifuged for 10 min at 200 g, the supernatant was removed, and the remaining pellet was subjected to 1.5 mL of 0.14% collagenase type I (Gibco, Waltham, USA) for 45 min, while it was later placed in incubator with 5% CO_2_ at 37 °C and 100% humidity. Five milliliters of DMEM-F12 containing 10% fetal bovine serum (FBS, Gibco, Waltham, USA) was added to the tube, and the content was spun again for 7 min at 200 g. The supernatant was removed, and the cell pellet was carefully re-suspended in 1 mL of media containing 88% DMEM-F12, 10% FBS, 1% penicillin-streptomycin, and 1% non-essential amino acids (Sigma-Aldrich, St. Louis, USA).

The suspended cells were transferred into a culture flask already containing 4 mL of DMEM-F12 (10% FBS, 1% penicillin-streptomycin, and 1% non-essential amino acids) and the culture flask was placed in a 5% CO_2_ incubator at 37 °C and 100% humidity. The media was changed every 3 days, until the cells were confluent. The cells were assessed morphologically under an inverted microscope (Nikon, Tokyo, Japan). Images were provided from cells using a digital camera (Olympus, Tokyo, Japan). Sub-culturing of cells was undertaken at 70% confluence by treating the cells with 0.25% (w/v) trypsin-EDTA (Gibco, Waltham, USA) until third passage.

### 2.3. Cell Characterization

For cell characterization, morphologic assessment, differentiation induction, and RT-PCR were undertaken. In the morphologic assessment, DPSCs (passage 3) were evaluated using an inverted microscope. To evaluate differentiation properties, the cells were assessed for osteogenic and adipogenic differentiation properties. For osteogenic induction, the DPSCs were transferred into 6-well plates to reach 70% confluence. The medium was replaced with osteogenic medium containing complete culture medium supplemented with 15% FBS, 100 nM dexamethasone (Sigma-Aldrich, St. Louis, USA), 50 µM ascorbic acid (Merck, Darmstadt, Germany), and 10 mM glycerol 3-phosohate (Merck, Darmstadt, Germany) for 3 weeks.

The media was changed every 2 days and after 3 weeks the cells were fixed with 10% formalin for 20 min, and, after several washes with deionized water, they were stained with fresh 1.4% Alizarin Red solution solved in deionized water at pH of 4.1 (Sigma-Aldrich, St. Louis, USA). For adipogenic differentiation, the DPSCs were plated in 6-well plates containing complete culture medium to reach 70% confluence. The medium was changed to adipogenic medium consisting of complete culture medium supplemented with 15% FBS, 100 nM dexamethasone, 100 µM ascorbic acid, and 200 μM of indomethacin (Sigma-Aldrich, St. Louis, USA) for 3 weeks. The cells were then fixed in 10% formalin for 20 min and, after several washes with deionized water, they were stained with fresh 0.5% Oil Red-O (Sigma-Aldrich, St. Louis, USA) solved in 2-propanol solution (Merck, Darmstadt, Germany) for 2 h.

The RT-PCR was done to determine the expression of 208 bp cluster of differentiation (CD) 73 (forward primer of TGCATCGATATGGCCAGTCC and reverse primer of AATCCATCCCCACCGTTGAC) and 177 bp CD90 (forward primer of GACCCAGGACGGAGCTATTG and reverse primer of TCATGCTGGATGGGCAAGTT) as mesenchymal markers; and 257 bp CD34 (forward primer of AGCCATGTGCTCACACATCA and reverse primer of CAAACACTCGGGCCTAACCT) and 450 bp CD45 (forward primer of CCAAGAGTGGCTCAGAAGGG and reverse primer of CTGGGCTCATGGGACCATTT) as hematopoietic markers. RNA extraction kit (Cinna Gen Inc., Tehran, Iran) was used to obtain the total RNA based on the manufacturer’s protocol. The first-strand cDNA was synthesized using the Revert Aid™ first strand cDNA synthesis kit (Thermo Fisher Scientific, Waltham, USA). The PCR thermal cycler (Veriti Thermal Cycler, Thermo Fisher Scientific, Waltham, USA) was applied for all PCR runs under the following plan: 1 cycle at 94 °C for 3 min, 35 cycles at 94 °C for 30 s, 60 °C for 30 s, and 72 °C for 30 s; and 1 cycle at 72 °C for 10 min; and, finally, electrophoresis was conducted and the bans were visualized.

### 2.4. MTT Assay

MTT assay was done to clarify the effect of SPIONs on proliferation rate of DPSCs. The cells were plated in 96-well plates (5000 cells/200 µl), while the media was changed after 24 h with either solution of (i) just culture media (control group), or (ii) SPIONs (Nanomag magnetic dextran nanoparticle, 130 nm particle size, Micromod Partikel Technologie GmbH, Rostock, Germany) at various concentrations added to the culture media (experimental group, 1.5, 2.5, 3.5, 4.5, 5.5, 12, and 25 mg/mL of SPIONs). It is necessary to mention that based on the Manufacturer’s protocol, iron oxide Chemical Abstracts Service (CAS) of 1317-61-9: 13 wt% and dextran CAS of 9004-54-0: 87 wt% were selected; while 3.5 mg/mL of SPIONs contained 327 µg/mL of iron based on atomic mass. Selection of 3.5 mg/mL dose of SPIONs as an optimum dose was based on (i) findings from previous studies [19], (ii) MRI of 1.5 T used in our study necessitating a high tracking dose, and (iii) the closest dose to the control with positive result.

After 24 h, 20 µL of a solution containing 5 mg/mL of dimethylthiazol-2-yl)-2,5-diphenyltetrazolium bromide, a tetrazole (MTT: Sigma-Aldrich, St. Louis, USA), was added to each well and incubated at CO_2_ incubator for 4 h at 37 °C and 100% humidity. The media was removed gently and, in order to dissolve the formazan crystals, 200 µL of DMSO per well (Merck, Darmstadt, Germany) was added. The cell viability was evaluated at optical density of 570 nm on a microplate reader (Floustar Omega, BMG Lab Tech, Ortenberg, Germany) (n = 4). The cell viability was calculated using the following formula of survival rate (%) = A sample − (Ab × AcAb) × 100; where Ac and Ab were considered as the absorbance in the control and blank wells, respectively.

### 2.5. Growth Kinetics

The growth kinetics of cells was evaluated by trypan blue exclusion method. The cell viability was assessed until 6 days using the cells labeled with 3.5 mg/mL of SPIONs (defined dose by MTT assay) cultured in 24-well plates (22,000 cells/well). To do so, 0.4% of trypan blue solution (Sigma-Aldrich, St. Louis, USA) was mixed with the cell suspension. The cells were observed and counted under a phase contrast microscope (Olympus, Tokyo, Japan) with a Neubauer hemocytometer slide. The population doubling time (PDT) was determined and the growth curve was illustrated using the defined formula, where T is the incubation time in h, Xb is the cell number at the beginning of the incubation time, and Xe is the cell number at the end of the incubation time, Ln = log_e_ and e = the Euler’s number.

(1) $$\mathrm{PDT}=\;T\times \frac{\ln2}{\ln\frac{\mathrm{Xe}}{\mathrm{Xb}}}N=\frac{n1+n2+n3+n4}{4}\times 2\times v\times 100.$$

### 2.6. Cell Labeling

DPSCs were labeled using 3.5 mg/mL of SPIONs for 48 h. These particles were shown to be separated with a permanent magnet and were prepared via the core-shell method with a core of magnetite and a dextran shell, consisted of 75%–80% (w/w) magnetite in a matrix of dextran (40,000 Da), and were conjugated with OH, NH_2_, and COOH groups for the covalent binding of proteins, antibodies, or other molecules as the Manufacturer described. Following the incubation, 3 × PBS washes were undertaken to remove excess particles. The DPSCs were later trypsinized, washed three times with PBS, and centrifuged at 300 g for 5 min.

The cells were fixed in 4% formalin at room temperature for 20 min and then centrifugation was performed for 5 min at 300 g. Following 3 × PBS washes, the cells were suspended in 200 µL of PBS and 200 µL 15% agarose gel (Sigma-Aldrich, St. Louis, USA). The groups were defined as 1 (DPSCs labeled with 3.5 mg/mL SPIONs), 2 (non-labeled DPSCs), 3 (15% gel), 4 (5 mg/mL SPIONs in 15% gel), 5 (15% gel), 6 (5 mg/mL SPIONs), 7 (DPSCs labeled with 3.5 mg/mL SPIONs), 8 (DPSCs labeled with 0.35 mg/mL SPIONs), 9 (H_2_O), and 10 (non-coated iron oxide particle).

### 2.7. In Vitro Assessment by MRI

For in vitro cell tracking by MRI, a clinical 1.5 T MRI scanner (General Electric, Signa HDXt, GE Healthcare, Chicago, USA) was used. The T2-weighted MR imaging parameters were slice thickness of 3 mm, repetition time of 460 ms, echo time of 24 ms, magnetic field strength of 1.5, flip angle of 25, matrix size of 512 × 512, and field of view of 220.01 mm. The SPIONs had a small crystal size. Therefore, the orientation of their lattice did not hinder their magnetic moments. The contrast agent significantly shortened the T2 relaxation time and this in turn led to the creation of a hypointense contrast on the spin-echo sequences. The SPIONs resulted in a larger hypointense contrast on the T2 gradient echo images. Hypointense areas of signal voids highlighted the existence of iron oxide-labeled cells.

### 2.8. Prussian Blue Staining

To assess in vitro efficiency of SPIONs uptake, Prussian blue staining was performed. To this end, the DPSCs were fixed in 10% formalin, washed 3 times by deionized water, and were finally subjected to 2 mL of a solution made of a 1:1 ratio of 20% aqueous solution of HCL (Merck, Darmstadt, Germany) and 10% aqueous solution of potassium ferrocyanide (Sigma-Aldrich, St. Louis, USA), and left at room temperature for 10 min. The cells were again washed 3 times in distilled water, and counterstained with nuclear fast red (Sigma-Aldrich, St. Louis, USA) for 5 min, and rinsed twice in distilled water. Xylazine and 95% ethanol (Merck, Darmstadt, Germany) were then used to visualize the SPIONs under a light microscope (Olympus, FSX100, Tokyo, Japan), as the presence of iron was visible as bright blue, cell nuclei in red, and cytoplasm appeared in pink.

### 2.9. Real-Time PCR

To quantify Bax and Bcl-2 gene expressions, DPSCs were harvested for total cellular RNA extraction 48 h after treatment of the cells with SPIONs using an RNA extraction kit (Cinna Gen Inc., Tehran, Iran). The quantity and quality of obtained RNA were checked by measuring the ratio of optical density (A_260_/A_280_ and A_260_/A_230_) using a Nanodrop™ spectrophotometer (Nanodrop; Thermo Fisher Scientific, Waltham, USA). The cDNA was synthesized using 1000 ng total RNA in a first-strand cDNA synthesis reaction using the Revert Aid™ first strand cDNA synthesis kit (Thermo Fisher Scientific, Waltham, USA).

The Bax and Bcl-2 genes were used as targets and B2m as an endogenous control. The sequences of interest genes were obtained from the NCBI database and primer sets were designed by primer3 software. Oligonucleotide sequences for 244 bp B2m (forward primer of CGTGCTTGCCATTCAGAAA and reverse primer of ATATACATCGGTCTCGGTGG), 134 bp Bcl-2 (forward primer of ATCGCTCTGTGGATGACTGAGTAC and reverse primer of AGAGACAGCCAGGAGAAATCAAAC), and 174 bp Bax (forward primer of CTGCAGAGGATGATTGCTGA and reverse primer of GATCAGCTCGGGCACTTTAG) were determined. RT-PCR was done using SYBR Green I as reporter dye and Step One Real-Time PCR reactions (Applied Biosystems, Waltham, USA). In each reaction, 200 nM of each primer was added to target the specific sequence. The PCR conditions were set for 10 min at 94 °C followed by 40 cycles of 15 s at 94 °C, 60 s at 60 °C, and melting curve analysis ramping from 65 to 95 °C. The amplification signals of different samples were normalized to B2m cycle threshold (Ct), and then the 2^-ΔΔCt^ method was used for comparing mRNA levels of underlying groups, which represented a fold-change in data analysis.

### 2.10. Flow Cytometry

Apoptosis analysis was done by flow cytometry. For both labeled and non-labeled DPSCs, 1 × 10^6^ cells were trypsinized and then transferred into a tube. Using a cold PBS solution, the cells were centrifuged at 200 g for 5 min, and washed twice with PE Annexin V apoptosis detection kit I (BD Biosciences, Franklin Lakes, USA) suspended in 0.1 mL of Annexin V 1X buffer. Harvesting of DPSCs was undertaken 48 h after treatment of cells with SPIONs. A total of 5 μL of PE Annexin V and 5 μL of 7-AAD was later added, slowly vortexed, and left for 15 min at room temperature. A total of 400 μL of Annexin V 1X as attachment buffer was added and read through the FACS Calibur™ flow cytometer (BD Biosciences, Franklin Lakes, Germany) during an hour and analyzed by the FlowJo software (BD Biosciences, Franklin Lakes, Germany). The amounts of early apoptosis were determined as the population percentage of Annexin V+/7-AAD- and late apoptosis as the population percentage of Annexin V+/7-AAD+ stained cells.

### 2.11. In Vivo Assessment by MRI

To trace SPION-labeled cells in vivo by MRI, the cells were trypsinized and then centrifuged at 300 g for 5 min. After 3 × PBS washes, intraperitoneal injection of 2 × 10^6^ DPSCs was undertaken. An in vivo MRI was performed for 2 recipient rats in supine position 2 h after intraperitoneal injection of both non-labeled and labeled DPSCs using a clinical 1.5 T MRI scanner (Siemens Magnetom Verio 3.0 T, Erlangen, Germany). The T2-weighted MR imaging parameters included slice thickness of 4 mm, repetition time of 420 ms, echo time of 24 ms, magnetic field strength of 1.5 T, flip angle of 25, width of 240.03 mm, and height of 512 mm. The rats were randomly allocated to experimental and control groups.

### 2.12. Statistical Analysis

For statistical analysis, the results were analyzed by one-way analysis of variance (ANOVA) using Prism version 6.0 software (GraphPad Software, San Diego, USA). A *p* value <0.05 was considered statistically significant.

## 3. Results

### 3.1. Cell Characterization

**Figure 1:**
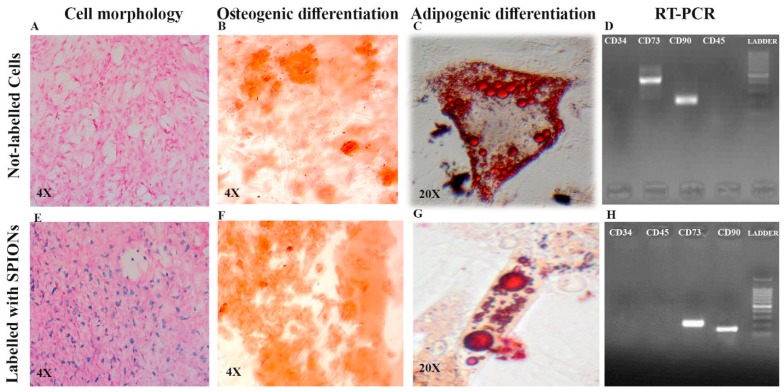
Comparison of cell morphology of dental pulp stem cells (DPSCs) ((**A**) non-labeled and (**E**) labeled DPSCs), osteogenic induction measurement using Alizarin Red staining ((**B**) non-labeled and (**F**) labeled DPSCs), adipogenic induction measurement using Oil Red-O staining ((**C**) non-labeled and (**G**) labeled DPSCs), and RT-PCR to characterize the cell differentiation ((**D**) non-labeled and (**H**) labeled DPSCs). Superparamagnetic iron oxide nanoparticles (SPIONs); cluster of differentiation (CD).

Both non-labeled and labeled DPSCs were adherent to the culture plates and fibroblast-like and had spindle-shape morphologies, respectively (Figure 1A,E). For osteogenic induction, non-labeled and labeled cells in osteogenic media demonstrated calcium deposition, revealed by Alizarin Red staining in the cells after three weeks (Figure 1B,F). Regarding adipogenic induction, non-labeled and labeled DPSCs stained by Oil Red-O also revealed intracellular lipid droplets in red color (Figure 1C,G). DPSCs showed positive expression of CD73 and CD90 and negative expression of CD34 and CD45 (Figure 1D,H).

### 3.2. MTT Assay

**Figure 2:**
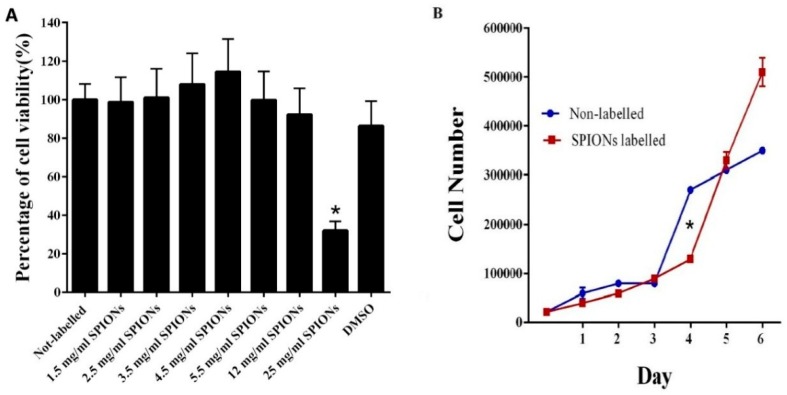
(**A**) MTT assay comparing the viability and proliferation capacity of different DPSCs. 1: Non-labeled cells, 2: Labeled cells with 1.5 mg/mL of SPIONs, 3: Labeled cells with 2.5 mg/mL of SPIONs, 4: Labeled cells with 3.5 mg/mL of SPIONs, 5: Labeled cells with 4.5 mg/mL of SPIONs, 6: Labeled cells with 5.5 mg/mL of SPIONs, 7: Labeled cells with 12 mg/mL of SPIONs, 8: Labeled cells with 25 mg/mL of SPIONs, 9: DMSO. The assay indicated that the SPIONs did not induce any significant decrease in cell viability at doses less than 25 mg/mL compared to non-labeled cells (mean ± SEM, * *p* < 0.05). **B:** The number of non-labeled and labeled DPSCs with 3.5 mg/mL of SPIONs.

MTT assay did not show any significant reduction in viability and proliferation capacity for labeled cells with SPIONs at doses less than 25 mg/mL, considered as IC_50_ = 15.494, in comparison to the control group (non-labeled cells) (Figure 2A). Figure 2B shows the number of non-labeled and labeled DPSCs with 3.5 mg/mL of SPIONs after six days, which indicates the absence of any significant statistical difference when DPSCs were treated with 3.5 mg/mL of SPIONs. The PDT for non-labeled and labeled DPSCs with 3.5 mg/mL of SPIONs after six days is shown in Table 1, denoting no significant statistical difference between them.

### 3.3. Prussian Blue Staining

In Prussian blue staining, the passive incubation period of DPSCs with SPIONs showed internalization of the particles within DPSCs, looking blue.

### 3.4. Real-Time PCR

**Figure 3:**
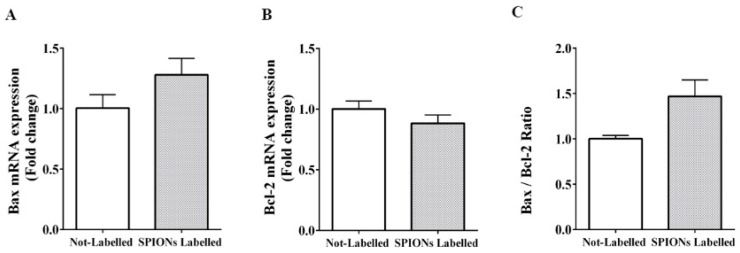
The effect of SPIONs on the expression level of the pro-apoptotic gene in labeled DPSCs assessed by RT-PCR ((**A**) Bax), anti-apoptotic genes ((**B**) Bcl-2), and Bax:Bcl-2 ratio (**C**) (mean ± SEM, no statistical difference was noted).

RT-PCR findings revealed that gene expression of the anti-apoptotic gene (Bcl-2) was 0.88-fold in labeled DPSCs compared to non-labeled cells. Additionally, Bax mRNA expression as a pro-apoptotic gene in labeled cells was 1.27-fold compared to non-labeled DPSCs. The ratio of Bax to Bcl-2 (Bax:Bcl-2) for labeled cells was determined as 1.44-fold compared to non-labeled DPSCs. No significant difference was noticed between experimental groups for Bcl-2 (*p* = 0.21), Bax (*p* = 0.14), and the ratio of Bax to Bcl-2 (Bax:Bcl-2) expression (*p* = 0.07) (Figure 3).

### 3.5. Flow Cytometry

**Figure 4:**
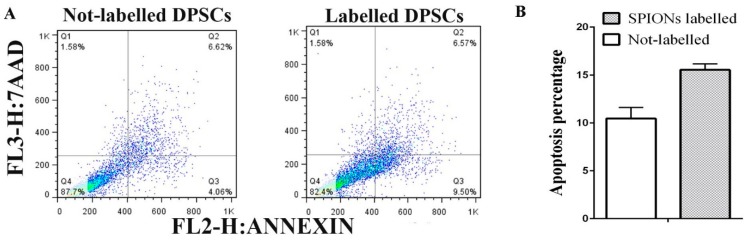
(**A**) Dead cells were scored as necrotic (Annexin V-negative/7-AAD-positive, upper left quadrants, Q1), late apoptotic (Annexin V-positive/7-AAD-positive, upper right quadrants, Q2), early apoptotic (Annexin V-positive/7-AAD-negative, lower right quadrants, Q3), and a gating on the normal cells that are considered viable, are PE Annexin V and 7-AAD negative (lower left quadrants, Q4). **B.** Apoptosis percentage was indicated as a bar chart (mean ± SEM, no statistical difference was noted).

Figure 4 shows that DPSC expression was negative for PE Annexin V and 7-AAD at the beginning of the apoptotic process. However, the expression was positive for PE Annexin V and 7-AAD in the final stages of apoptotic process and towards the cell death. The flow cytometry results also showed that SPION-labeled DPSCs showed 5% increase in the apoptosis (Annexin V+/7-AAD+ positive expression).

### 3.6. In Vitro Assessment by MRI

**Figure 5:**
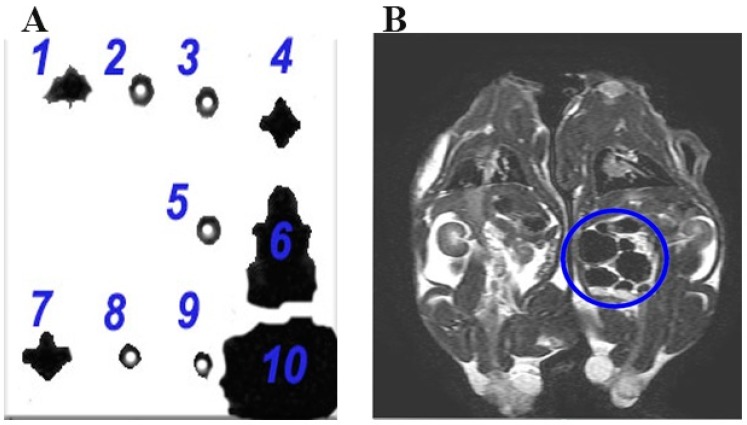
**A:** The T2-weighted MRI of in vitro samples. 1: DPSCs labeled with 3.5 mg/mL of SPIONs showing a hypointense signal of the iron oxide contrast, 2: Non-labeled DPSCs without any hypointense signal for the iron oxide contrast, 3: 15% agarose gel lacking the hypointense signal of the iron oxide contrast, 4: 5 mg/mL of SPIONs in 15% gel revealing the hypointense signal of the iron oxide contrast, 5: 15% gel without any hypointense signal of the iron oxide contrast, 6: 5 mg/mL SPIONs demonstrating the hypointense signal of the iron oxide contrast, 7: DPSCs labeled with 3.5 mg/mL SPIONs denoting the hypointense signal of the iron oxide contrast, 8: DPSCs labeled with 0.35 mg/mL SPIONs in absence of any hypointense signal of the iron oxide contrast, 9: H_2_O lacking the hypointense signal of the iron oxide contrast, and 10: Non-coated iron oxide particle exhibiting an extremely hypointense signal of the iron oxide contrast. (**B**) The T2-weighted images of DPSCs labeled with 3.5 mg/mL SPIONs injected intraperitoneally with hypointense signal of the iron oxide contrast, in the right picture, compared to non-labeled DPSCs, in the left picture, lacking the hypointense signal (rats in supine position).

Figure 5A shows the T2-weighted MRI of samples in vitro. The hypointensity showed an increase with cell number and SPION concentration. The T2 eff was shorter for higher numbers of labeled cells. MRI detection thresholds were set at a T2 eff value of 0.084 ms. The SPIONs-labeled cells resulted in a T2 eff value of 0.084 ms or less by MRI, demonstrating that the T2 eff was in a lower value than anticipated (<5 ms). A greater loss in signal over a greater area was noted in the non-labeled cells tracked by MRI.

### 3.7. In Vivo Assessment by MRI

After intraperitoneal injection of DPSCs, MRI tracking confirmed the presence of SPIONs in labeled DPSCs in the peritoneal cavity of the rat in supine position (dark area), when compared to presence of non-labeled DPSCs in the control animal in the identical position. The T2-weighted images of DPSCs labeled with 3.5 mg/mL SPIONs injected intraperitoneally revealed a hypointense signal of the iron oxide contrast, when compared to non-labeled DPSCs lacking the hypointense signal (Figure 5B).

## 4. Discussion

Significant progress has been done in regenerative medicine using stem cells. However, the underlying mechanisms of using these cells in tissue repair still remain elusive due to the lack of effective, non-invasive, non-toxic, and clinically-acceptable modality for cell tracking after transplantation. Current approaches for cell tracking include direct radiolabeling, reporter genes for optical and radioactive imaging, and magnetic labeling of cells. MRI has been introduced as a non-invasive and efficient tool which is able to monitor migration and integration of cells after transplantation with a high resolution. SPIONs have attracted extensive interest due to their large surface area, high reactivity, small size, and high drug-loading capacity [20]. SPIONs have also been widely used as a imaging contrast agent in MRI for therapeutic and diagnostic applications [21]. Moreover, dextran coating of SPIONs enhances their efficiency for MRI tracking, as dextran-coated nanoparticles were shown to be taken up by cells through endocytosis [22]. These findings can explain why DPSCs labeled with dextran-coated SPIONs were efficiently tracked and detected by MRI.

SPIONs are composed of a magnetite (Fe_3_O_4_) or maghemite (γ-Fe_2_O_3_) iron oxide core that are usually inert and have been coated with several biocompatible polymers to facilitate cell tracking using MRI post-transplantation [16]. We showed the incorporation of SPIONs into DPSCs using Prussian blue staining. High efficiency of Prussian blue staining was shown previously to confirm the internalization of SPIONs in DPSCs [16,23,24]. SPIONS are the only metal nanoparticles receiving approval from the Food and Drug Administration [25]. They have little or no negative effect on survival, viability, proliferation, apoptosis, stemness, and function of cells, which make them a valuable component for cell transplantation [23,26,27,28,29,30]. Additionally, no inflammatory response was reported for SPIONs when they were used for diagnosis of myocardial infarction and myocarditis. After intravenous administration, these nanoparticles were taken up by activated monocytes and macrophages, which were predominantly accumulated in regions associated with inflammation [31].

MRI confirmed the intracellular uptake of dextran-coated SPIONs by cells when injected intraperitoneally, in which the viability, proliferation, and functionality of the labeled cells were not affected at doses less than 25 mg/mL of SPIONs [32]. The intraperitoneal injection of labeled DPSCs was just a pilot study to document presence of SPIONs in vivo to be a base for further studies using SPIONs-labeled cells in regenerative medicine in animal models.

Iron oxide labeling caused apoptosis in endothelial cells and macrophages [33]; however, not in progenitor or stem cells [2,34]. In our study, we labeled DPSCs with SPIONs and did not observe any cytotoxicity and proliferation, and functionality of the labeled cells were not influenced by labeling based on the results from MTT assay, growth kinetics, RT-PCR, and flowcytometry, except for doses higher than 25 mg/mL.

There are some controversies about effect of SPIONs on differentiation of labeled cells. Some researchers showed an inhibitory effect of magnetic nanoparticles on chondrogenic differentiation of stem cells [23], but others demonstrated that labeled stem cells were able to differentiate into chondroblasts [35,36]. Some studies reported differentiation ability of MSCs after SPION labeling [23,36]. However, Chen et al. showed an inhibitory effect of SPIONs on osteogenic induction of MSCs [37]. Our results showed positive osteogenic and adipogenic induction of MSCs labeled with magnetic nanoparticles.

In our study, the size of SPIONs was 130 nm and 1.5 T MRI was used. Some researchers reported that SPIONs efficiently incorporated stem cells in a dose-dependent manner [38]. To yield the greatest contrast to track and visualize labeled cells, it is important to use more efficient MRI scanners, such as 1.5 T [16,39] and 3 T scanners [16,38], and tune SPION size and concentration. We aim to do further studies to reveal the effect of MRI intensity and SPIONs physical characteristics on DPSC detection in vivo, to be a platform for further in vivo studies using stem cells labelled with SPIONs. We found that MRI was a non-invasive method for tracking of transplanted cells labelled with SPIONs that can replace biopsies as invasive sampling methods. Time-course tracking for presence of SPIONs in DPSCs was not undertaken in the in vitro assessment and, in a qualitative study, the correlation of SPIONs and the signal loss in the MRI image was investigated.

Various cell types have been labeled with SPIONs and have been visualized in vitro and in vivo using MRI [40]. Noorwali reported the therapeutic role of bone marrow-derived MSCs labelled with SPIONs in the amelioration of hepatic injury/cirrhosis in rats [41]. We used DPSCs because they have mesenchymal properties and, in the recent decade, have opened a window in regenerative medicine based on their anti-inflammatory, immunomodulating, and healing properties. They can easily be prepared in dental clinics by non-invasive methods from patients seeking orthodontic treatment or tooth extraction or from falling teeth in children, while stem cells extracted from bone marrow and adipose stem are recovered by invasive methods [10].

In vivo tracking of stem cells using SPIONs has already been extensively studied in several animal models. For example, labeled MSCs were tracked in liver having hepatocellular carcinoma using MRI [42]. In another study, after transplantation of SPION-labeled MSCs in rats via the portal veins, they were visualized by MRI showing the improvement in the hepatic pathology and diminishing the cirrhosis [43]. Bos et al. used MRI for tracking SPIONs-labeled MSCs transfused into the renal arteries and portal veins without any hazardous effect on the viability and differentiation of the injected cells [44]. Lee et al. successfully used MRI to track ferumoxytol-labeled MSCs [45]. It was also shown that MSCs labeled with MRI-visible nanoparticles reduced the infarct volume of stroke [46]. Delcroix et al. reported, for the first time, that neural migratory pathways can be established and mapped out by MRI using SPIONs with no effect on the morphology or viability of the MSCs [47]. Some authors reported in vivo MR imaging of labeled cells in osteochondral defects up to 4–12 weeks using magnetic nanoparticles with a diameter between 11 and 150 nm [35,48,49]. In a pilot in vitro and in vivo study to detect drug-induced apoptosis using Annexin V-conjugated ultra-small superparamagnetic iron oxide, their potential for detection of drug-induced apoptosis was confirmed [50]. These studies confirmed our successful tracking of DPSCs after intraperitoneal injection in the animals that can be a platform for further in vivo studies using stem cells labelled with SPIONs.

## 5. Conclusions

We provided the in vitro evidences supporting the feasibility of an MRI-based method to monitor DPSCs labeled with dextran-coated SPIONs without any significant reduction in viability and proliferation capacity, and differentiation properties of labeled cells showing that internalization of SPIONs within DPSCs were not toxic at doses less than 25 mg/mL. Therefore, MRI can stand as a non-invasive method to track stem cells labeled by SPIONs both in vitro and in vivo. SPIONs are biocompatible, easily available, and cost effective, opening a new avenue in regenerative medicine.

## Figures and Tables

**Table 1:** Comparison of population doubling time (PDT) between non-labeled and SPION-labeled DPSCs.

	Groups	Non-labeled DPSCs (h:min)	SPION-labeled DPSCs (h:min)
Day			
Day 1		16:57	28:54
Day 2		26:13	33:54
Day 3		39:19	35:59
Day 4		26:46	37:55
Day 5		31:42	30:56
Day 6		36:21	31:58

Dental pulp stem cells (DPSCs); superparamagnetic iron oxide nanoparticles (SPIONs).
